# Bone Marrow Edema and Tyrosine Kinase Inhibitors Treatment in Chronic Myeloid Leukemia

**DOI:** 10.3390/diagnostics15243112

**Published:** 2025-12-08

**Authors:** Sabina Russo, Manlio Fazio, Giuseppe Mirabile, Raffaele Sciaccotta, Fabio Stagno, Alessandro Allegra

**Affiliations:** Division of Hematology, Department of Human Pathology in Adulthood and Childhood “Gaetano Barresi”, University of Messina, Via Consolare Valeria, 98125 Messina, Italy; dottoressarusso20@gmail.com (S.R.); manliofazio@hotmail.it (M.F.); giuseppe.mirabile@polime.it (G.M.); sciaccottaraffaele@gmail.com (R.S.); aallegra@unime.it (A.A.)

**Keywords:** bone marrow edema, chronic myeloid leukemia, tyrosine kinase inhibitors, bosutinib, CML toxicities, CML therapy, magnetic resonance imaging, platelet-derived growth factor receptor α and β

## Abstract

**Background and Clinical Significance:** Tyrosine kinase inhibitors (TKIs) have transformed Philadelphia chromosome-positive chronic myeloid leukemia (Ph+ CML) into a largely manageable chronic disease. However, off-target toxicities are increasingly recognized; rarer complications such as bone marrow edema (BME) remain underreported. BME is a radiological syndrome characterized by excess intramedullary fluid on fat-suppressed T2/STIR magnetic resonance imaging sequences and may progress to irreversible osteochondral damage if unrecognized. We report a case series of TKI-associated BME and propose a practical diagnostic-therapeutic framework. **Case Presentation**: We describe three patients with Ph+ CML who developed acute, MRI-confirmed BME of the lower limb during TKI therapy. Case 1 developed unilateral then bilateral knee BME, temporally associated first with dasatinib and subsequently with imatinib; symptoms improved after TKI interruption, bisphosphonate therapy, and supportive measures, and did not recur after switching to bosutinib. Case 2 presented with proximal femoral BME during long-term imatinib; imatinib was stopped, intravenous neridronate administered, and bosutinib initiated with clinical recovery and later near-complete radiological resolution. Case 3 experienced multifocal foot and ankle BME during imatinib; symptoms resolved after drug discontinuation and bisphosphonate therapy, and disease control was re-established with bosutinib without recurrence of BME. All patients underwent molecular monitoring and mutational analysis to guide safe therapeutic switching. **Discussion:** Temporal association across cases and the differential kinase profiles of implicated drugs suggest PDGFR (and to a lesser extent, c-KIT) inhibition as a plausible mechanistic driver of TKI-associated BME. PDGFR-β blockade may impair pericyte-mediated microvascular integrity, increase interstitial fluid extravasation, and alter osteoblast/osteoclast coupling, promoting intramedullary edema. Management combining MRI confirmation, temporary TKI suspension, bone-directed therapy (bisphosphonates, vitamin D/calcium), symptomatic care, and, when required, therapeutic switching to a PDGFR-sparing agent (bosutinib) led to clinical recovery and preservation of leukemia control in our series. **Conclusions:** BME is an underrecognized, potentially disabling, TKI-related adverse event in CML. Prompt recognition with targeted MRI and a multidisciplinary, stepwise approach that includes temporary TKI adjustment, bone-directed therapy, and consideration of PDGFR-sparing alternatives can mitigate morbidity while maintaining disease control. Prospective studies are needed to define incidence, risk factors, optimal prevention, and management strategies.

## 1. Introduction

A current clinical challenge in Philadelphia positive-chronic myeloid leukemia (Ph+ CML) is the early detection of adverse events to both prevent intolerance induced by TKIs therapy with long-term sequelae and preserve treatment efficacy. For example, in recent years, considerable attention has been directed toward the cardiovascular and glycol-metabolic adverse events of some TKIs (especially nilotinib and ponatinib), inducing hematology and cardiology societies to issue dedicated guidelines on cardiovascular management in CML patients undergoing TKI therapy [1,2,3]. On the other side, real-world medical practice often allows clinicians to encounter some underrecognized or rare toxicities that deserve better definition and clearer interpretation [4]. To this address, we report our experience of bone marrow edema (BME) developing during dasatinib (DAS) and imatinib (IM) treatment. When not promptly recognized, BME can progress to disabling and potentially irreversible sequelae, such as avascular necrosis, subchondral insufficiency fractures, or degenerative joint disease [5]. We aim to highlight this potentially underreported adverse effect of *BCR::ABL1* TKIs therapy and to propose a rational framework for the therapeutic management of those CML patients who develop BME during TKI therapy. We further combined our clinical observations with a review of the literature. A detailed search was conducted using the PubMed and Scopus databases. Boolean operators such as “AND,” “OR,” and “NOT” were applied to refine results, using combinations of keywords including “bone marrow edema,” “chronic myeloid leukemia,” “tyrosine kinase inhibitors,” “dasatinib,” “imatinib,” “bosutinib,” “adverse events,” and “PDGFR inhibition.” Filters were applied to include articles published in English, with emphasis on clinical trials, case reports, and reviews from the last 15 years.

## 2. General Considerations for Bone Marrow Edema

### 2.1. Definition of Bone Marrow Edema

BME is a condition characterized by the abnormal accumulation of fluid within the trabecular bone tissue, affecting the knee joint, causing spontaneous pain and tenderness on palpation [6]. This phenomenon reflects an inflammatory or microvascular process that leads to increased intraosseous pressure and associated bone pain. BME can occur in various conditions, including infections, degenerative processes, or drug-induced toxic effects [7,8,9,10,11]. In addition, preexisting pathologies such as inflammatory diseases or neoplasms may lead to BME by triggering several interlinked mechanisms. In inflammatory conditions (e.g., rheumatoid arthritis, synovitis, or metabolic bone diseases), immune and stromal cells produce pro-inflammatory cytokines (like IL-1β, IL-6, and TNF-α), which increase vascular permeability within the subchondral bone and bone marrow. This causes fluid leakage, increased intraosseous pressure, and decreased perfusion. In neoplastic cases, tumor infiltration or bone marrow metastases disrupt normal bone marrow architecture, weaken vessel walls, and induce local ischemia or microvascular compression. Taken together, these inflammatory and neoplastic processes create nonspecific osteochondral reactions, including reactive bone turnover, BME, and eventually damage to cartilage if unresolved [12]. Lastly, bone marrow lesions (BML) associated with BME, although frequently resolving spontaneously, may sometimes exacerbate preexisting osteoarthritic changes and may lead to joint replacement surgery [13]. Radiologically, BME appears as hyperintense areas on fat-suppressed T2-weighted, short tau inversion recovery (STIR), or proton density magnetic resonance imaging (MRI) scans, reflecting intramedullary fatty replacement by water-containing tissue components. Histologically, lesions show lymphocytes’ infiltration, hypervascularization, bone demineralization, and tissue fibrosis, indicating a more complex etiology underlying BME [14].

### 2.2. Etiopathogenesis of Bone Marrow Edema

The etiopathogenetic mechanisms underlying BME are multifactorial [14]. Both chronic microtrauma and acute traumatic events play a pivotal role in BME development by altering intramedullary vascular permeability, promoting fluid and blood retention in the marrow space. Specifically, dysregulated microcirculation, arising from either excessive perfusion or impaired blood flow, has been implicated in the pathogenesis of BME formation by increasing intraosseous pressure (IOP) and further compromising local vascularization [15]. The increased intraosseous pressure and resulting tissue hypoxia can irritate nerve endings, causing the characteristic pain of this condition. Pro-inflammatory and proalgesic substances, such as prostaglandins, critically contribute to tissue damage and amplify pain by stimulating nociceptors [16,17]. These pathogenetic mechanisms can be classified into three principal categories: (1) ischemic; (2) mechanical; and (3) reactive (Table 1).

### 2.3. Diagnosis of Bone Marrow Edema

MRI is the gold standard for localizing acute and chronic lesions of the articular cartilage and subchondral bone, allowing early identification of BME at symptom onset [30]. It should be mentioned that dual-energy CT (DECT) has gained recognition as a useful alternative, since it offers shorter acquisition times, reduced costs, and greater availability in emergency settings compared with MRI. It also supports timely decision-making when MRI is impractical or contraindicated [30]. On MRI, subchondral bone alterations appear as ill-defined hypointense areas on T1-weighted images and hyperintense areas on fat-suppressed T2-weighted or STIR sequences acquired with Fast Spin Echo (FSE). In particular, T1WI is useful for distinguishing benign BME-like changes from marrow replacement processes, such as neoplasms or osteomyelitis. According to the Society of Skeletal Radiology, the signal intensity on T1WI remaining higher than that of muscle or intervertebral disk is generally consistent with benign BME-like signal. By contrast, isointense or hypointense T1 signal reveal suspicion for marrow replacement. In such cases, additional techniques, including Dixon in-phase and out-of-phase imaging, can assist in differentiating benign lesions from malignant infiltration or infection [31]. After intravenous contrast administration, increased signal intensity can be observed at the affected sites, indicative of increased local vascularization and bone remodeling [32,33]. Based on formation mechanism and location, lesions can involve not only subchondral bone but even ligaments; consequently, BMLs are traditionally classified as subchondral or ligamentous [34]. Trauma-associated forms frequently involve meniscal and ligamentous structures [35]. Subchondral lesions are observable in early stages of knee osteoarthritis and may serve as potential early markers of the disease. Cartilage erosion is commonly seen in osteoarthritis and subchondral lesions, underscoring their close pathological correlation [36]. The strong association between BML and cartilage damage highlights their role in osteochondral degeneration [36]. Moreover, MRI evaluations over 24 months show progressive signal increase, especially at the medial tibial plateau and medial femoral condyle, reflecting cartilage degeneration [37].

### 2.4. Bone Marrow Edema Therapy

Therapeutic approaches to BME should be tailored according to whether the condition follows a self-limiting course or shows progression signs. Differential diagnosis remains crucial in defining causative factors and appropriate treatment approaches [38]. The self-limiting nature of BME often warrants conservative management initially, including limb offloading for 3–6 weeks, combined with anti-inflammatory, analgesic, and physiotherapeutic treatments [39]. Nonsteroidal anti-inflammatory drugs (NSAIDs), including acetylsalicylic acid (ASA), play a multifaceted role in BME treatment. Beyond the well-known analgesic and anti-inflammatory effects, ASA offers antiplatelet activity through irreversible inhibition of cyclooxygenase-1 (COX-1), thereby reducing thromboxane A2 synthesis and platelet aggregation. This mechanism not only helps to manage pain and inflammation but also contributes to vascular endothelial regulation, improving microcirculation within the bone marrow compartment. In BME, ASA-therapy helps in modulating vascular tone, reducing platelet activation, and supporting tissue recovery and symptom relief. These properties make ASA a valuable adjunct in conservative treatment protocols, especially in cases with suspected ischemic or reactive vascular components [6]. Bisphosphonates (alendronate, ibandronate, pamidronate, neridronate, and clodronate) play a key role in BME treatment by reducing osteoclastic activity and preventing subchondral bone collapse. Monitoring vitamin D and calcium levels during bisphosphonate therapy is essential, with supplementation, as needed, to reduce hypocalcemia risk [40]. Furthermore, it is necessary to prevent possible long-term complications, such as osteonecrosis of the jaw [41]. Given the role of prostaglandins in BME, analogs such as Iloprost have demonstrated efficacy, especially in post-ischemic and post-traumatic BME, by improving intramedullary microcirculation. According to this, Zippelius et al. have recently published a meta-analysis of eleven studies evaluating the efficacy of intravenous Iloprost therapy in patients with BME syndrome of the proximal femur. In these series, the pathogenesis of BME was related to local microvascular dysfunction and impaired bone perfusion (leading to intraosseous hypertension and ischemia-induced bone marrow changes) and not to drug-related mechanisms. More than 90% of patients reported symptom relief and/or showed an MRI-confirmed edema reduction. Pain improvement often occurred within the first week of treatment, and only a small subset (3.2%) required surgical intervention following therapy. In general, despite variability in dosing protocols and staging criteria across studies, Iloprost consistently showed a benefit, particularly in early stages (I-II) of the Association Research Circulation Osseous (ARCO) staging system, and may be considered [42]. Certain physical therapies, like extracorporeal shock wave therapy (ESWT), have shown effectiveness in BME, as mechanical waves improve regional blood flow and reduce edema [43]. More specifically, the mechanical pressure waves generated by ESWT induce microtrauma, which stimulates angiogenesis and vasodilation, thereby improving regional blood microcirculation and reducing intraosseous pressure. This vascular response is particularly beneficial in congestive BMES, where vascular occlusion, often drug-induced, is a key pathogenic factor. In traumatic BMES, ESWT promotes bone tissue repair and remodeling by enhancing the expression of bone morphogenetic proteins (BMPs) such as BMP-2, BMP-3, BMP-4, and BMP-7, which are essential for osteogenesis and fracture healing. This regenerative effect helps reinitiate the bone remodeling cycle, especially in cases of trabecular microfractures [43]. Surgical intervention is typically indicated when conservative treatment fails, especially in lesions larger than 5 cm^2^ or involving over 50% of the condyle, which includes core decompression and drilling into the spongy bone to reduce intraosseous pressure and alleviate pain [44].

## 3. Case Presentation

The clinical scenario of CML has significantly changed since the introduction of TKIs [45]. In most cases, long-term treatment and molecular monitoring are required [46], with life expectancy approaching that of the general population [47] and allowing us to achieve a deep molecular response in a substantial number of patients [47]. However, despite these outstanding results, few data are reported on BME as an adverse event alongside TKI treatment [48,]. The association between the first case of BME and treatment with TKI (DAS) was identified belatedly, and, as in 2021, neither the drug’s technical datasheet nor the literature reported such a correlation. Clinical management is challenging and might impact significantly on the QoL of the patients with CML. Here, we report a series of three patients affected by chronic myeloid leukemia who developed bone marrow edema during treatment with tyrosine kinase inhibitors. CML clinical management and molecular monitoring of *BCR::ABL1* transcript levels were performed according to the European Leukemia Network (ELN) criteria. Diagnosis and monitoring of BME were primarily based on magnetic resonance imaging (MRI), as described previously.

**CASE 1: A CML patient under dasatinib and imatinib treatment**

**Figure 1:**
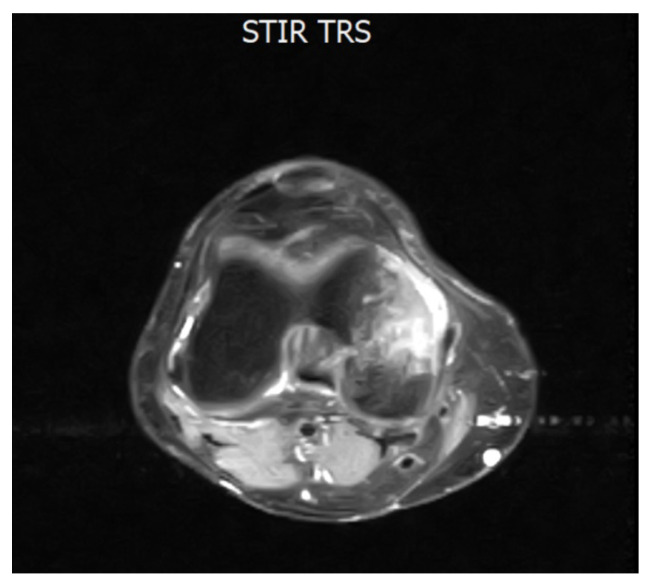
Axial MRI section of the knee showing intra-spongious bone marrow edema of the medial femoral condyle. The hyperintense signal is visible on STIR and T2 fat-saturated sequences, consistent with marrow edema. This image dates to February 2021, when the patient completed 28 months of TKI treatment with DAS and referred to a sudden onset of joint pain. Abbreviations: STIR (short tau inversion recovery) and TRS (tool rotation speed).

A 43-year-old Caucasian male was diagnosed with CML, Ph+, e14a2 *BCR::ABL1* transcript with a low prognostic risk (Sokal 0.56, ELTS 1.1675), with disease onset in July 2018. He had no comorbidities. His family history was negative for oncological diseases but positive for cardiovascular diseases. First-line treatment with DAS, a second-generation (2G) TKI, was initiated at standard dose (100 mg daily) and the patient soon achieved an optimal molecular response (MR3). After 22 months of treatment, the dose was reduced to 80 mg/day due to the onset of two episodes of pleural effusion, which were both reversible with corticosteroid therapy at low-dose, diuretics, and short DAS interruptions. However, six months later, despite a maintained MR3, the patient developed sudden pain in the right knee joint, resulting in significant impairment of ambulation. Walking was only possible with support (crutch-assisted). MRI of the right knee and lower limb showed marked intra-spongious BME in the medial femoral condyle, along with adjacent soft tissue edema [Figure 1].

**Figure 2:**
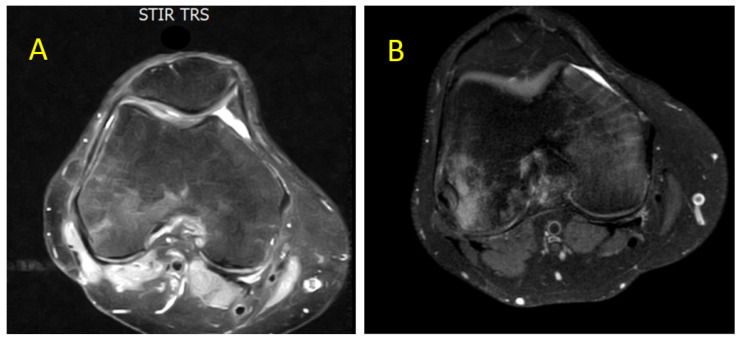
Axial MRI section of the knee showing gradual and progressive improvement of previously documented intra-spongious BME of the medial femoral condyle. (**A**) Partial resolution of BME after 2 months of laser therapy, TECAR therapy, bisphosphonates, and NSAIDs (April 2021). (**B**) Further improvement of BME after 4 months associated with clinical relief of bone-related symptoms (June 2021). Both images were acquired using STIR and T2-weighted fat-saturated sequences. ABBREVIATIONS: NSAIDs (nonsteroidal anti-inflammatory drugs) and TECAR (capacitive and resistive electric transfer).

Clinical orthopedic evaluation led to a diagnosis of idiopathic BME. Given the patient’s significant functional impairment, DAS therapy was discontinued to allow for treatment with NSAIDs and resolution of the inflammatory process. The patient was also prescribed strict rest and bisphosphonates (standard-dose clodronate). Following gradual symptom improvement (about 10 days), DAS was reintroduced at 80 mg/day. According to orthopedic recommendations, physiotherapy, capacitive and resistive electric transfer (TECAR) therapy, and laser therapy were continued in combination with bisphosphonates and NSAIDs. After approximately one month, clinical improvement was noted with reduced pain. Within two months, ambulation was restored without support, along with improved MRI findings (April 2021) [Figure 2A]. Due to a new episode of pleural effusion, DAS was further reduced to 50 mg/day, while bone edema treatments were continued. By June 2021, MRI showed further improvement [Figure 2B].

**Figure 3:**
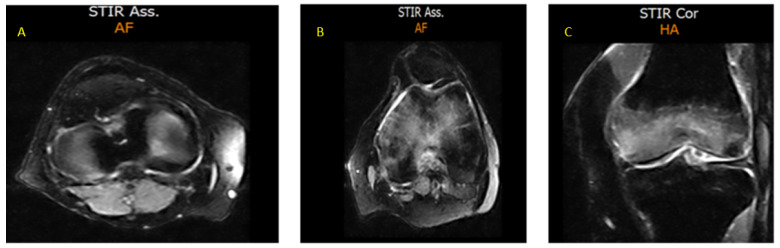
MRI sections of both knees, documenting BME (February 2022). (**A**) Axial STIR section of the right knee showing a hyperintense intra-spongious signal within the medial femoral condyle, consistent with localized BME; (**B**) Axial STIR section of the left knee demonstrating a more extensive hyperintense area of intra-spongious BME, affecting the medial femoral condyle and spreading towards the subchondral region, without associated cortical disruption; (**C**) Coronal STIR section of the left knee which further highlights the patchy, ill-defined margins of the BME, with a distribution typical of mechanical overload or subchondral stress reaction.

The patient continued DAS 50 mg/day with a sustained MR3 response and no recurrence of knee pain. However, on month 36 of TKI treatment (December 2021), a further episode of pleural effusion led to permanent DAS discontinuation. After resolution of the pulmonary event, re-evaluation confirmed stable MR3 and no detectable mutations. The patient refused to switch to a third generation TKI because of cardiovascular risk concerns. Therefore, he was switched to IM (first generation TKI) as second-line therapy at a conventional dose (400 mg quotidie). In February 2022, after only 8 days on IM therapy, the patient developed acute bilateral knee pain with consequent immobility. MRI confirmed bilateral BME [Figure 3A–C].

**Figure 4:**
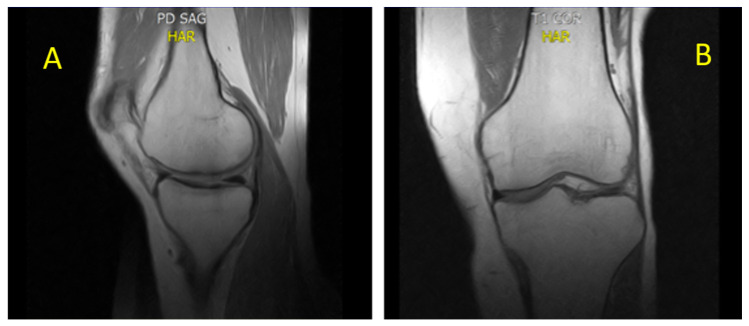
(**A**) sagittal proton density and (**B**) coronal T1. Sagittal proton density (PD) and coronal T1-weighted MRI of the knee, demonstrating complete resolution of BME (November 2022). The trabecular bone exhibits a homogeneous, well-compacted appearance, characterized by uniformly high signal intensity on T1-weighted sequences and the absence of hyperintense areas on PD-weighted images. The cortical margins appear intact and sharply defined, with restoration of normal bone architecture and signal distribution consistent with reestablished marrow integrity.

Considering the rapid development of bilateral BME following IM initiation, IM was permanently discontinued. The patient was referred to the orthopedic department where he began a specific bisphosphonate regimen: intravenous neridronate 100 mg diluted in 500 mL of saline weekly for four infusions, followed by intramuscular neridronate 25 mg every 15 days for 6 months. Supportive therapy included cholecalciferol (initially 1000 IU/day orally, then 25,000 IU weekly; later adjusted to every 15 days based on lab and imaging monitoring) and calcium (600 mg/day orally), along with anti-inflammatory medications, resulting in gradual symptom relief. MRI reassessment performed in November 2022 demonstrated complete resolution of the previously detected BME with restoration of normal trabecular architecture and signal intensity [Figure 4A,B]. However, after nearly two months of TKI discontinuation, molecular monitoring revealed the loss of MR3, thus necessitating the resumption of leukemia treatment. Based on the hypothesis that the BME could be linked to PDGFR inhibition, third-line therapy with bosutinib (BOS) was started, since BOS does not target PDGFR [49]. Therefore, the patient began BOS at a low dose (100 mg/day), which was increased weekly to 400 mg/day, achieving an optimal molecular response (MR3) again without recurrence of BME and without requiring supportive therapy for BME. The patient is alive and on BOS 400 mg daily treatment, maintaining a stable MR3 response.

**CASE 2: 60-year-old patient with imatinib therapy**

**Figure 5:**
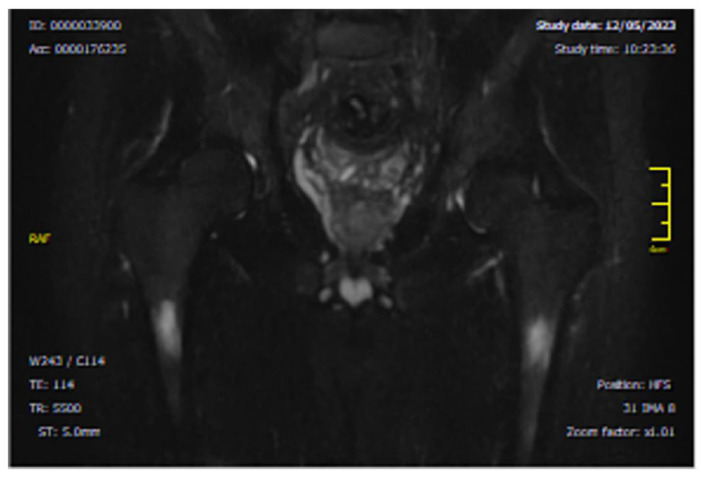
Coronal MRI section, showing bilateral intra-spongious BME of the femoral diaphysis. The image shows a focal bilateral hyperintense area on STIR sequence (T2-weighted with fat suppression) in the femoral diaphysis.

A 60-year-old Caucasian male was diagnosed with CML Ph+, e14a2 *BCR::ABL1* transcript, intermediate prognostic risk according to Sokal (0.88) and low ELTS score (1.0188), in July 2021, at the age of 58. His medical history included bilateral maculopathy, coxarthrosis with narrowing of the coxofemoral joint space and early acetabular sclerosis, hyperhomocysteinemia, and a negative family history for oncological diseases whilst a positive family history for cardiovascular diseases. First-line treatment with IM was initiated at the standard dose of 400 mg daily, resulting in a deep and sustained molecular response (MR4) achieved and maintained from the sixth month of therapy, together with a good drug tolerance. Only minor interruptions occurred, due to infections and one episode of grade 2 hematologic toxicity [Common Terminology Criteria for Adverse Events (CTCAE): leukopenia]. On month 22 (May 2023), the patient experienced a sudden onset of left hip joint pain with severe gait impairment, requiring crutches. Initially, suspecting an exacerbation of pre-existing coxarthrosis, IM was suspended to allow for analgesic and anti-inflammatory therapy. MRI of the left hip reported no joint abnormalities but focal areas of altered signal intensity in the proximal femoral diaphysis, hypointense on T1-weighted images and hyperintense on STIR sequences, with associated mild edema of the adjacent bone marrow [Figure 5].

Based on these radiological features, we excluded the hypothesis of a merely degenerative origin, since no sign of worsening of the pre-existing arthrosis was found. We have also contemplated the hypothesis of progression to coxo-arthritis [13], but the images did not show markedly reactive features or condral collapse. Therefore, considering that the BME was acute, focal, and appeared de novo during TKI therapy, an iatrogenic origin was suspected.

**Figure 6:**
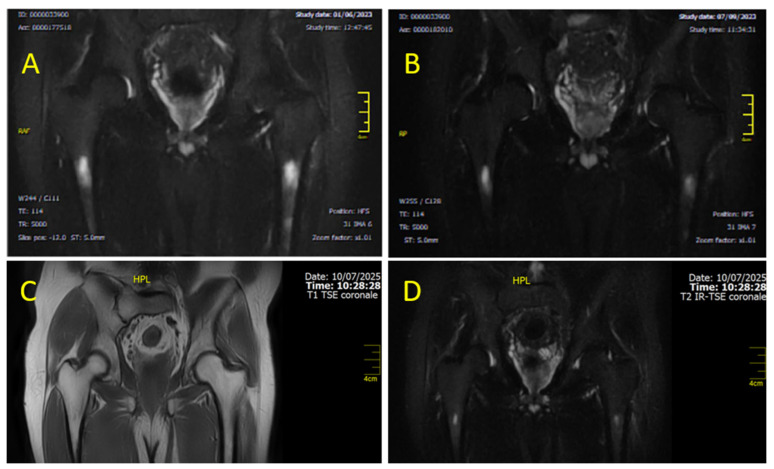
Coronal MRI sections of the femoral diaphysis illustrate the temporal evolution of BME over time. Image (**A**), acquired in June 2023 using STIR (T2-weighted fat-suppressed) sequences, shows stable BME with no significant interval changes compared to previous assessments. However, image (**B**) (T2W), obtained a few months later (September 2023), already demonstrates a clear reduction in both the extent and intensity of the edema. Images (**C**) (T1-weighted) and (**D**) (T2-weighted fat-suppressed) correspond to the follow-up MRI performed in July 2025, demonstrating further regression of BME and restoration of normal marrow signal. At this stage, the patient persists, clinically asymptomatic and free of bone-related complaints.

Accordingly, IM was permanently discontinued and treatment with bisphosphonates, vitamin D, and calcium was initiated. After completing four intravenous neridronate infusions, the patient showed a rapid clinical improvement. On 7 June 2023, just 22 days after the radiological diagnosis of BME, criteria for second-line treatment eligibility for CML were confirmed. BOS therapy was initiated at 300 mg daily. Over the following three months, gradual resolution of hip pain and complete functional recovery were observed, although the imaging findings remained unchanged [Figure 6A,B]. In September 2023, because of a decrease in molecular response to MR2, BOS therapy increased to 400 mg/day. This allowed the patient to regain a deep molecular response through six months of therapy. The patient has continued treatment with BOS at 400 mg/day with good tolerance and is maintaining a deep molecular response. At the most recent follow-up on July 2025, coronal T1- and T2-weighted MRI sequences [Figure 6C,D] demonstrated only minimal residual BM signal alteration, which is consistent with near-complete resolution of BME. This latest outcome further strengthens the suspect of an iatrogenic cause, rather than a consequence of a pre-existing degenerative affection (which, by definition, can be restrained but not reverted). The patient remains asymptomatic, with full functional recovery and sustained QoL.

**CASE 3: 66-year-old patient with imatinib therapy**

A 66-year-old Caucasian female diagnosed with Ph+ CML, e13a2 *BCR::ABL1* transcript, with intermediate Sokal (0.73) and low ELTS (1.1883) scores, was diagnosed in August 2016 at the age of 58. Her medical history included estrogen-receptor-positive breast cancer (treated in 2007 with quadrantectomy, axillary lymphadenectomy, adjuvant chemotherapy, and radiotherapy), thyroid nodules, chronic obstructive pulmonary disease (COPD), tricuspid valve insufficiency, dyslipidemia, arterial hypertension, anxiety-depressive disorder, colonic diverticulosis, and colitis. At diagnosis, the patient had multiple medications: antihypertensives (bisoprolol), lipid-lowering agents (atorvastatin), bronchodilators, thyroid hormone replacement (levothyroxine), anxiolytics and antidepressants (benzodiazepines), proton pump inhibitors (pantoprazole), and occasional use of NSAIDs. IM treatment commenced as first-line therapy at 400 mg/day. During the first 24 months, the patient experienced frequent interruptions due to drug intolerance (periorbital and peripheral edema, infections, diarrhea, and lower limb cramps). Consequently, major molecular response (MR3) was achieved only by month 30 of treatment. However, persistent toxicity (periorbital and lower limb cramps and edema) and severe pain, particularly in the left ankle, prompted treatment discontinuation. The pain resolved upon stopping IM. Persistent lower limb pain, despite absence of peripheral edema (lower limb Doppler ruled out peripheral vascular disease), raised the suspicion of BME. MRI performed in November 2023 showed: “Bone marrow edema signal hyperintensity in the calcaneus, anterior talus, and tarsal bones, especially the cuneiforms (possible algodystrophy)”. Therefore, IM was permanently discontinued, and standard BME treatment with bisphosphonates (as per previous cases) was initiated. The patient showed rapid clinical improvement, with pain resolution and return to normal ambulation being approximately one month after diagnosis. However, MRI findings remained unchanged. In parallel, CML molecular monitoring revealed an increase in *BCR::ABL1* transcript levels (*BCR::ABL/ABL1^IS^*: 10.44%). Sanger mutational analysis was negative. Hence, treatment with BOS at 300 mg/day was initiated. A gradual dose escalation was attempted, but the patient experienced multiple toxicities (cutaneous, laboratory abnormalities, and recurrent infections). Despite interruptions, the dose was increased to 400 mg/day after six months, with moderate tolerance. By month 10 of treatment, MR3 molecular response was regained. Since then, the patient has continued treatment with BOS at 400 mg/day with fair pharmacological tolerance and maintenance of MR3, with no further episodes of bone edema.

## 4. Discussion

The therapeutic management of CML represents a complex and multifaceted field, which is heavily influenced by the specific and nonspecific target effects of the drugs. These agents can significantly impact patients’ QoL: either improving or worsening it. The clinical cases presented herein highlight this complexity and underscore the need for an approach that is not exclusively hematological but multidisciplinary. The association between the use of a TKI and the onset of an iatrogenic adverse effect might not be immediately identifiable. Indeed, several confounding factors often might be involved. Among TKI-induced adverse events, several lines of evidence suggest a correlation of the BME onset with the use of *BCR-ABL*-targeted TKIs. For instance, BME is listed as a rare adverse effect in the technical documentation of DAS. Additionally, a case of BME was described in a patient affected by GIST and treated with IM: discontinuation of the drug led to a regression of bone symptoms, suggesting a potential adverse drug reaction [50].

### 4.1. From Clinical Observation to Mechanistic Hypothesis

The first patient proved to be particularly paradigmatic, as he developed BME during treatment with both DAS and IM. This observation prompted us to hypothesize a potential overlapping of the pharmacological actions of these two TKIs. Notably, both agents are known to inhibit platelet-derived growth factor receptor (PDGFR) [51], suggesting a shared pathophysiological mechanism underlying the onset of BME. BME may result from a cumulative pharmacological effect, whereby prolonged DAS exposure might progressively disrupt bone remodeling and vascular integrity through sustained PDGFR inhibition. The transient improvement observed, despite ongoing therapy, likely reflects the beneficial impact of a multimodal supportive strategy (including anti-inflammatory drugs, bisphosphonates, and rehabilitative interventions such as physiotherapy, TECAR, and laser therapy). Moreover, the progressive dose reduction (from 100 mg to 80 mg, and later to 50 mg/day) may have attenuated PDGFR inhibition, allowing for a partial restoration of bone homeostasis while maintaining molecular control of leukemia. Finally, the unilateral nature of the initial episode, precipitated by mechanical strain, highlights the possible interplay between pharmacologically induced vulnerability and localized biomechanical stress, suggesting that individual susceptibility factors modulate the clinical expression of TKI-associated BME. Altogether, this first case was instrumental in shaping the hypothesis on the role of PDGFR in the pathogenesis of TKI-related bone complications and in guiding the mechanistic interpretation discussed in the following paragraphs. Regarding the other two cases, alternative causes, such as degenerative joint disease or comorbid conditions, were evaluated. As specified in the case 2 description, no MRI signs of progressive arthrosis or arthritis development were reported. As a consequence, we have excluded these options and pursued the theory of a drug-induced manifestation. Regarding case 3, although this patient presented with multiple comorbidities and polypharmacy, none of the concomitant conditions or medications are known to induce focal BME. Regarding her comorbidities, she never experienced a breast cancer relapse and thyroid tests were within ranges; regarding polypharmacy, though PPIs reduce calcium uptake and favor bone turnover and statin may, on the contrary, contribute to bone formation (enhancing osteoblast activity [52]), no specific features of bone resorption or bone remodeling were found. The other comorbidities and therapies have no significative connection with the skeletal apparatus. Therefore, considering the temporal association with long-term TKI exposure and improvement after dose adjustment, a TKI-related mechanism appears plausible. Notably, the other concomitant therapies were not suspended nor reduced.

### 4.2. The Role of PDGFR-β in Imatinib-Induced Edema Formation

IM is a selective tyrosine kinase inhibitor targeting *BCR-ABL1*, *KIT*, and *PDGFRB*, and was the first drug designed to act directly on its molecular target. Following the landmark IRIS trial in 2002, IM became the standard first-line therapy for newly diagnosed CML, achieving hematologic response rates up to 98% and complete cytogenetic responses in 65–85% of patients. It has also shown short-term efficacy in the blastic phase of CML and is widely used in treating gastrointestinal stromal tumors (GIST) [53]. While generally well-tolerated, IM is associated with mild-to-moderate side effects in fewer than 10% of cases, including muscle cramps, myelosuppression, gastrointestinal symptoms, skin reactions, and localized edema (e.g., periorbital or pretibial). Most fluid retention responds to conservative measures, but rare and more severe complications, such as optic disk edema, intramuscular edema, pleural or pericardial effusions, intra-articular fluid accumulation, and cerebral edema have been reported [54,55]. The pathophysiology behind IM-induced edema remains incompletely understood, but growing evidence implicates its potent inhibition of *PDGFR-β*. Platelet-derived growth factor receptor-β (*PDGFR-β*) plays a central role in preserving vascular integrity and regulating interstitial fluid homeostasis through its actions on vascular mural cells, including pericytes and vascular smooth muscle cells. Under physiological conditions, activation of *PDGFR-β* by its ligands (PDGF-BB in particular) is crucial for pericyte recruitment and survival. Pericytes stabilize endothelial cells by forming intimate contacts along the capillary wall, where they regulate endothelial barrier function, basement membrane synthesis, and capillary contractility [56]. This pericyte–endothelial cross talk limits vascular leakage by strengthening endothelial junctions and reducing paracellular permeability. In addition, *PDGFR-β* signaling helps maintain interstitial pressure. Through pericyte-mediated regulation of the extracellular matrix and modulation of interstitial hydrostatic forces, *PDGFR-β* prevents excessive extravasation of plasma fluid into the interstitium [56]. This is partly mediated via *PI3K/Akt* and MAPK-dependent pathways, which promote cell survival, regulate cytoskeletal dynamics, and preserve the structural integrity of microvessels [57]. When *PDGFR-β* activity is inhibited (as occurs with TKIs like imatinib), pericyte coverage of capillaries is reduced, endothelial junctions loosen, and interstitial pressure falls: all of which facilitate fluid extravasation and edema formation [58]. Preclinical studies using transgenic, PDGFR-β-knockout mice have demonstrated that the loss of this marker results in diminished pericyte coverage and pronounced impairments in vascular integrity, underscoring its essential role in preserving microvascular architecture, avoiding excessive endothelial permeability [59]. Although these data are derived primarily from muscle tissue, endothelial systems, and angiogenesis assays, the underlying mechanisms (pericyte dropout, vascular leakage, and impaired interstitial pressure regulation) are biologically relevant to the bone microenvironment and offer a coherent explanatory framework for the BME observed in our patients. Future work should extend these insights by evaluating similar pathways in preclinical models of drug-induced BME and, in experimental systems, using targeted *PDGFR-β* inhibition (e.g., nilotinib, sorafenib, sunitinib, crenolanib), which would help to clarify the contribution of this signaling axis to TKI-associated skeletal complications. Renal mechanisms also contribute, as imatinib interferes with tubular sodium handling, leading to water and salt retention and promoting generalized edema. In some patients, impaired lymphatic drainage further decreases pleural fluid clearance, amplifying effusion development. Finally, hypoalbuminemia observed in selected cases lowers plasma oncotic pressure, favoring fluid shifts from the intravascular compartment into the interstitial and pleural spaces. Together, these processes could explain how IM, although generally associated with mild periorbital or peripheral swelling, may occasionally cause severe pleural and pericardial effusions, particularly in patients on long-term or higher-dose therapy [60].

### 4.3. PDGFR- and c-KIT-Mediated Modulation of the Osteochondral Unit Under TKI Therapy

*PDGFR-α/β* and *c-KIT* are present on both osteoblasts and osteoclasts and act as essential receptors involved in bone cell regulation. Focusing on these cellular elements, osteoblasts are mesenchymal-derived cells which are fundamental in maintaining bone health: they synthesize the bone matrix and promote its mineralization. Their dysfunction is implicated in various inflammatory and sclerosing skeletal disorders [61]; osteoclasts are hematopoietic-derived multinucleated cells. These cells are essential for bone homeostasis: they resorb the mineralized bone matrix and regulate skeletal remodeling. Their dysfunction contributes to metabolic bone diseases and pathological bone loss, such as osteoporosis and inflammatory osteolysis [62]. Recent investigations have demonstrated that sustained therapy with TKIs (such as IM and DAS) may disrupt the osteochondral unit by targeting these very receptors expressed on both osteoblasts and osteoclasts [61]. In addition, further emerging evidence focuses on the role of both *PDGFRα* and *β* in osteoclastogenesis regulation, thus contributing to the ultimate definition of *PDGFR* signaling as a master modulator of bone homeostasis [63]. Interestingly, the inhibition of *PDGFR* signaling induced by imatinib leads to two opposing mechanistic effects. On one side, it enhances osteoblast differentiation and mineralization; on the other side, it simultaneously restrains the proliferation of osteoblast precursors and suppresses osteoclastogenesis through both direct actions on precursors and indirect stromal cell-mediated pathways [61,64]. Regarding these latter pathways, IM reduces the release of key paracrine factors such as macrophage colony-stimulating factors (M-CSF), receptor activator of nuclear factor kappa-B ligand (RANKL), and vascular-endothelial growth factor (VEGF), which are essential for osteoclast precursor survival, differentiation, and the coupling of bone remodeling with angiogenesis [65]. Because of this dual activity in the bone microenvironment, IM may contribute to the increase in trabecular bone volume and density, and at the same time induce a paradoxical site-specific reduction in bone mineral density (particularly at the femoral neck) [65]. DAS shares similar off-target effects, as demonstrated by the enhanced osteoblast differentiation and decreased osteoclast viability in vitro [66]. In total, these findings underscore the dual osteoanabolic and antiresorptive actions of TKIs, potentially explaining how long-term inhibition of *KIT* and *PDGFR* pathways may lead to abnormal bone remodeling and altered skeletal homeostasis in patients treated with these drugs.

### 4.4. From Muscle Edema to Bone Marrow Edema: Vascular Effects of TKIs

While musculoskeletal adverse effects from DAS are relatively uncommon, affecting roughly 6% of patients, with severe symptoms in <1% of cases, instances of muscle edema have been reported and are believed to stem from its inhibition of *PDGFR-β* (key regulator of vascular integrity) [67]. This mechanism yields compelling parallels with the pathogenesis of BME, wherein impaired *PDGFR-β* signaling may similarly disrupt interstitial fluid regulation and promote fluid extravasation in bone tissues. Further supporting this analogy, an investigation into DAS-induced vascular effects revealed that this drug compromises the integrity of the endothelial barrier and the regulation of angiogenesis (which are both essential for maintaining fluid homeostasis). This effect may plausibly drive localized fluid accumulation in muscle and BM [68]. The same mechanism can be extended to other similar adverse manifestations, such as pleural effusion. In fact, the simultaneous appearance in the first patient of both BME-related symptoms and a pleural effusion further strengthens the link between these adverse events and *PDGFR* inhibition [69]. Lastly, in the third clinical case, periodic IM discontinuation due to toxicity was accompanied by improvement in bone symptoms, adding another piece to the understanding of this issue. Recent experimental work on *BCR::ABL1* protein stability has highlighted how selective disruption of kinase-dependent multiprotein complexes can alter tissue homeostasis and microenvironmental signaling. In preclinical systems, *BCR::ABL1* destabilization, triggered by perturbations in its oligomeric structure and downstream effectors such as *PI3K* subunits, *SHIP2*, *Sts1*, and *Shc*, reveals how small changes in kinase conformation or interaction networks can influence cellular stress responses, cytoskeletal dynamics, and local cytokine output. Although these findings were obtained under thermal stress rather than pharmacologic inhibition, they illustrate a broader principle: differences in TKI binding profiles may translate into distinct tissue responses, including altered endothelial permeability, modified stromal–vascular interactions, extracellular matrix remodeling, and perturbations in local fluid handling. This framework reinforces the plausibility that agents with specific off-target activity, such as *PDGFR-β* inhibition, may predispose to localized BME by transiently affecting microvascular integrity or stromal signaling [70].

### 4.5. Therapeutic Switching to Bosutinib: Clinical and Mechanistic Justification

In this context, the therapeutic switch to BOS was not only clinically justified but also mechanistically sound. Unlike IM and DAS, BOS does not inhibit *PDGFR* signaling, thereby sparing a receptor pathway that plays a central role in vascular integrity, interstitial fluid regulation, and bone homeostasis. This pharmacological distinction reinforces the hypothesis that the *PDGFR* blockade is a key driver in the pathogenesis of iatrogenic BME. The subsequent resolution of marrow edema following the transition to BOS lends further support to this causal link. Mechanistically, by preserving *PDGFR*-mediated pericyte recruitment and endothelial stabilization, BOS avoids the microvascular dysfunction and dysregulated interstitial fluid dynamics associated with *PDGFR* inhibition. Moreover, its lack of activity against *PDGFR* minimizes off-target interference with the osteoblast and osteoclast signaling pathways, reducing the risk of skeletal remodeling abnormalities. Taken together, these observations highlight BOS as a safer therapeutic option for patients developing musculoskeletal or bone marrow fluid-related toxicities on earlier-generation TKIs, while also underscoring the pivotal role of *PDGFR* in the pathophysiology of TKI-induced BME [71,72,73]. Bosutinib’s main safety liabilities are gastrointestinal (diarrhea, nausea, vomiting), transaminase elevations, and thrombocytopenia. Management is well-established: give with food and adequate hydration to mitigate diarrhea; use antidiarrheal agents early (e.g., loperamide) and consider dose interruption/reduction for persistent grade ≥ 2 toxicity; monitor liver enzymes monthly for the first 3 months and more frequently as clinically indicated; and apply dose modification or temporary interruption for transaminase elevations. Bosutinib exposure is strongly affected by CYP3A modulators (inhibitors increase exposure; inducers decrease exposure), so concurrent prescription of strong CYP3A inhibitors/inducers requires dose adjustments or avoidance [74].

An important practical consideration is that several *PDGFR*-sparing therapeutic options exist and each carries a distinct mechanistic and toxicity profile that must be weighed when selecting a switch strategy. Asciminib is a first-in-class allosteric inhibitor that binds the *ABL* myristoyl pocket and selectively inhibits *BCR::ABL1* without targeting *PDGFR*, making it an attractive mechanistic alternative when *PDGFR* inhibition is suspected to underlie toxicity [75]. Asciminib was originally approved for patients with chronic-phase Ph+ CML after failure or intolerance to ≥2 prior TKIs and/or for those harboring the *T315I* mutation [75,76]; more recent regulatory decisions have expanded its role into earlier lines. Mechanistically, because asciminib does not inhibit *PDGFR*, it avoids the peri-vascular/pericyte effects attributed to the *PDGFR-β* blockade and therefore is less likely to produce the microvascular leakage hypothesized to contribute to BME. Clinically, however, asciminib is associated with its own toxicity spectrum (notably cytopenias, elevations in pancreatic enzymes/pancreatitis, and hypertension) and has clinically relevant transporter-mediated drug–drug interactions (for example with some statins) [75,77]. Therefore, concurrent use of interacting drugs should be reviewed and adjusted, as indicated. In practice, choice among PDGFR-sparing options should be individualized: asciminib may be preferred for patients with the *T315I* mutation or in whom its safety profile and interaction plan are acceptable (e.g., when cytopenia risk and pancreatitis risk are manageable and drug–drug interactions can be mitigated), and BOS may be preferable when rapid disease control is needed and gastrointestinal (GI) toxicities can be managed. In our experience, none of the three patients harbored a *T315I* mutation or had contraindications related to the GI tract; therefore, to maintain therapeutic consistency across cases and ensure *PDGFR*-sparing TKI selection, BOS was chosen as the preferred agent.

## 5. Suggested Diagnostic/Therapeutic Algorithm and Conclusions

With more than twenty-five years in clinical use, tyrosine kinase inhibitors (TKIs) have dramatically changed the natural history of Ph+ CML [1,78]. Since the initial characterization of the disease in the 1960s, TKIs have been the first agents able to turn Ph+ CML into a treatable condition, with disease-related survival curves now being comparable to those of the general population [79,80,81]. Although successful in most cases, a proportion of the patients will experience either intolerance to or failure of treatment. In years of clinical practice, we have progressively learned how to recognize, manage, and mitigate safety issues; however, some of them may still substantially impact quality of life (QoL), therapeutic adherence, efficacy, and outcomes. In this regard, the identification of a possible rarely described side effect such as iatrogenic BME raises important questions regarding the optimal therapeutic management of patients with CML. As shown, multidisciplinary collaboration among personalized therapeutic strategies, such as switching to BOS, are fundamental elements to establish more effective pathways of care. CML patients undergoing TKI therapy who report persistent bone pain, especially in the lower limbs, deserve careful evaluation for the possibility of BME onset. To overcome this challenge, we propose the following diagnostic/therapeutic algorithm, structured in five steps:

(1) Diagnostic Confirmation: Perform targeted MRI to confirm or exclude the presence of BME;
(2) Initial Therapeutic Adjustment: Temporary discontinuation of the ongoing TKI if BME is confirmed.
(3) Supportive Bone-Directed Therapy: Bisphosphonates in combination with vitamin D and calcium supplementation. This timely approach allows us to enhance skeletal stability, stabilize the skeletal microenvironment, and counteract secondary bone metabolic alterations.
(4) Symptom Control: Associate symptomatic management with NSAIDs when clinically indicated to improve QoL.
(5) Long-Term Disease Management: Therapeutic switch to BOS following clinical resolution, ideally guided by mutation analysis and ongoing molecular monitoring to ensure both safety and clinical hematologic efficacy, according to actual guidelines.

Such an approach balances the need for continued disease control with the equally important goal of mitigating iatrogenic complications. Furthermore, early identification of BME allows for the prompt initiation of specific treatment, avoiding a prolonged suspension of CML therapy and reducing the risk of disease progression. It is also reasonable to hypothesize that the true incidence of BME may be underestimated. In fact, some cases may remain undiagnosed because patients are switched to alternative TKIs for other reasons or reach treatment-free remission, thereby resolving their symptoms without resuming the initial therapy. In such scenarios, musculoskeletal complaints may be addressed by orthopedic specialists, and since the previous TKI therapy is not reintroduced, a recurrence of BME does not occur, further masking its prevalence. Furthermore, future in vivo studies are needed to identify potential predictive biomarkers of BME risk, with the aim of guiding therapeutic choices from the first line and deepening the understanding of the pathogenesis of this disorder.

## Figures and Tables

**Table 1:** Major pathogenetic causes of BME: ischemic, mechanical, and reactive. Prominent characteristics associated with each subtype. ABBREVIATIONS: BME (bone marrow edema); MRI (magnetic resonance imaging); SCD (sickle cell disease); MPN (myeloproliferative neoplasms); ACL (anterior cruciate ligament); SPONK (spontaneous knee osteonecrosis); SONK (secondary knee osteonecrosis); PAONK (post-arthroscopic knee osteonecrosis); and CRPS (complex regional pain syndrome).

BME Pathogenesis	Cause	Characteristics	Ref
ISCHEMIC	Reduced blood perfusion	Knee osteonecrosis: • Spontaneous (SPONK) [18] - Older patients (>55 years) • Secondary (SONK) [18,19] - Younger patients (20 to 55 years) - Risk factors: alcohol abuse, obesity, corticosteroid use or specific clinical conditions (like SCD, MPNs, and Gaucher’s disease) • Post-arthroscopic (PAONK) [18] - Post-meniscectomy and chondroplasty BME syndrome[20]: • Typical MRI BME findings • Painful • Reduced joint mobility • Increased interstitial fluid Osteochondritis dissecans[21]: • Idiopathic (80% pain during weight bearing) • Focal subchondral bone disorder • Primarily affecting adolescents • Unstable osteochondral fragments • Associated osteoarthritis Algodystrophy or Morbus-Sudeck syndrome [22]: • Complex regional pain syndrome (CRPS) • 1/3 of pts evolve to chronicity • Presenting with: - Persistent burning pain - Trophic changes - Sensory disturbances	[6]
MECHANICAL *“Bone bruise ”*	Mainly traumatic events [Acute/chronic trauma and repeated stresses may lead to a breakdown of the marrow trabeculae, with interstitial fluid leakage and hemorrhage to BM spaces]	Most common form. It includes the following: Bone contusions [23]: • Trabecular microfractures caused by impact, traction, or direct trauma [e.g., acute ACL rupture, transitory lateral patellar dislocation, and varus and valgus injuries] Overload injuries [24]: • Subchondral bone overload typically associated with joint malalignment or mechanical stress Stress fractures [25]: • Fatigue fractures: repetitive overload of normal bone structures (runners, athletes) • Insufficiency fractures: spontaneous, without any trauma or overload, develop in a pathologic bone (e.g., osteoporosis)	[6]
REACTIVE	Inflammatory response to adjacent pathological processes	Generally associated with the following: • Osteomyelitis: patients often present systemic symptoms like fevers or sepsis [26] • Inflammatory arthritis (in the final stages): joint effusion, subchondral edema, geodes, and reactive synovitis [27] • Tumors: osteosarcoma, chondrosarcoma, and Ewing sarcoma [28] • Prior surgical interventions [29]	[6]

## Data Availability

Data related to this study are included in the manuscript.

## Abbreviations

The following abbreviations are used in this manuscript:

TKIs	Tyrosine Kinase Inhibitors
Ph+/-	Philadelphia Chromosome-Positive/Negative
CML	Chronic Myeloid Leukemia
QoL	Quality of Life
ELN	European Leukemia Network
BME	Bone Marrow Edema
MRI	Magnetic Resonance Imaging
STIR	Short Tau Inversion Recovery
DAS	Dasatinib
IM	Imatinib
*BCR::ABL1*	Breakpoint Cluster Region::Abelson Murine Leukemia Viral Oncogene Homolog 1
DECT	Dual-Energy Computed Tomography
T1(2)WI	T1/T2-Weighted Imaging
FSE	Fast Spin Echo
RQ-PCR	Real-Time Quantitative Polymerase Chain Reaction
NSAIDs	Non-Steroidal Anti-Inflammatory Drugs
BML	Bone Marrow Lesion
ASA	Acetylsalicylic Acid
COX-1	Cyclooxygenase-1
ARCO	Association Research Circulation Osseous
ESWT	Extracorporeal Shock Wave Therapy
BMP	Bone Morphogenetic Protein
MR3	Major Molecular Response (3-log reduction)
MR4	Deep Molecular Response (4-log reduction)
CTCAE	Common Terminology Criteria for Adverse Events
BOS	Bosutinib
IU	International Units
Sokal	Sokal Prognostic Score
ELTS	EUTOS Long-Term Survival Score
GIST	Gastrointestinal Stromal Tumor
PDGFR-α/β	Platelet-Derived Growth Factor Receptor
PI3K	Phosphoinositide 3-Kinase
MAPK	Mitogen-Activated Protein Kinase
M-CSF	Macrophage Colony-Stimulating Factor
RANKL	Receptor Activator of Nuclear Factor Kappa-B Ligand
VEGF	Vascular Endothelial Growth Factor
KIT	Stem Cell Factor Receptor (also known as CD117)
